# Racial and Regional Disparities in Trends in NICM-Related Mortality in the U.S. From 1999 to 2020

**DOI:** 10.1016/j.jacadv.2024.101083

**Published:** 2024-07-11

**Authors:** Olayinka J. Agboola, Niya A. Jones, Jared A. Spitz, Palak Shah, Jamie LW. Kennedy, Vanessa Blumer, Shashank S. Sinha, Garima S. Sharma

**Affiliations:** a St. Mary’s Hospital/Yale School of Medicine, Waterbury, Connecticut, USA; b Inova Schar Heart and Vascular, Inova Fairfax Medical Campus, Falls Church, Virginia, USA; c University of Maryland, Baltimore, Maryland, USA

Nonischemic cardiomyopathy (NICM) is the leading indication for heart transplantation in the United States, accounting for more than 50% of all heart transplantation.^1^ Our understanding of the etiology and pathophysiologic mechanisms underlying NICM has improved, with a concomitant increase in the therapeutic options available for this patient population.^2^ While overall survival among patients with NICM has improved, the literature continues to show sex and racial disparities in access to advanced heart failure treatments such as ventricular assist devices and heart transplantation.^3^^,^^4^ This study examined the overall temporal trend in NICM-specific age-adjusted mortality rates (AAMRs) and its variations based on patient sex, race, rurality, and geographical location.

We analyzed publicly available, anonymized, AAMR data from 1999 to 2020 obtained through the Center for Disease Control and Prevention's WONDER (Wide-ranging Online Data for Epidemiologic Research) database. The WONDER multiple-cause death file contains the single underlying cause of death data and up to 20 additional causes of death obtained from death certificates issued across the United States and coded by the National Center for Health Statistics staff based on International Classification of Diseases-10th edition codes. NICM was defined by International Classification of Diseases-10 codes I42.0 through I42.9.

AAMR, expressed as the number of deaths per 100,000 persons, was plotted against time (by year). We stratified the yearly AAMR by sex, race, census region, and urban/rural status and plotted this over time (by year) to determine trend variations within the groups. Joinpoint regression analysis was used to determine trends in AAMR expressed as an average annual percentage change (AAPC). Data were analyzed using SAS, version 9.4 and Joinpoint Analysis Software. Standardized rate ratio (SRR) was computed by direct calculation to compare AAMR between groups.^5^ The study was exempt from Institutional Review Board review and oversight.

**Figure 1:**
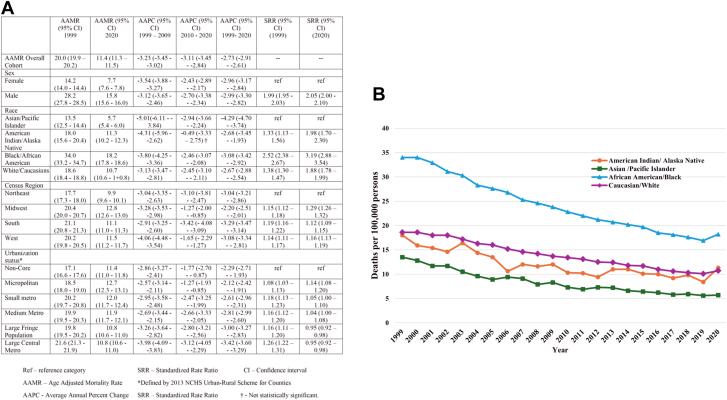
Disparities in Trends in Nonischemic Cardiomyopathy-Related Mortality (A) Age-adjusted mortality rate by group; (B) graph of age-adjusted mortality rate by race.

During the study period, a total of 1,062,066 NICM-related deaths occurred in 6,746,356,647 at-risk persons. There was a decline in the AAMR from 1999 (AAMR 20.0; 95% CI: 19.9-20.2) to 2020 (AAMR 11.4; 95% CI: 11.3-11.5) at an AAPC of −2.73% (95% CI: −2.91 to −2.61). This same decline was seen in both males and females from 1999 to 2020, with an AAPC of −2.99% (95% CI: −3.30% to −2.82%) in males and −2.96% (95% CI: −3.17% to −2.82%) in females. However, AAMR remained persistently higher in males. In 1999, the AAMR was 28.2 (95% CI: 27.8-28.5) for males and 14.2 (95% CI: 14.0-14.4) for females. In 2020, the AAMR for males was 15.8 (95% CI: 15.6-16.0) and for females was 7.7 (95% CI: 7.6-7.8). The SRR was 1.99 (95% CI: 1.95-2.03) in 1999 and 2.05 (95% CI: 2.00-2.10) in 2020 (Figure 1A).

Mortality was highest among African Americans and lowest among Asian/Pacific Islanders. This finding remained persistent throughout the study period. In 1999, African Americans had an AAMR of 34.0 (95% CI: 33.2-34.7) and Asian/Pacific Islander had AAMR of 13.5 (95% CI: 12.5-14.4) with an SRR of 2.52 (95% CI: 2.38-2.67). In 2020, the AAMR for African Americans was 18.2 (95% CI: 17.8-18.6) while Asian/Pacific Islanders had an AAMR of 5.7 (95% CI: 5.4-6.0) with an SRR of 3.19 (95% CI: 2.88-3.54). There was a consistent overall decline in AAMR from 1999 to 2020 in all four race categories. However, this decline had stalled significantly among American Indians/Alaska Natives (AI/AN) between 2010 and 2020, with an AAPC of −0.49% (95% CI: −3.33 to 2.75) (Figure 1B).

There was a significant overall downward trend in mortality across all four census regions and urbanization strata. However, the rate of decline was not homogenous, and there were significant regional variations. Between 2010 and 2020, the rate of decline decreased in the Midwest (1999-2009 AAPC of −3.28% [95% CI: −3.53 to −2.98] vs 2010 to 2020 AAPC of −1.27% [95% CI: −2.00 to −0.85]) and West (1999-2009 AAPC of −4.06% [95% CI: −4.48 to −3.54] vs 2010 to 2020 AAPC of −1.65% [95% CI: −2.29% to −1.27%]) census regions. Also, the decline in rural areas, that is, micropolitan (1999-2009 AAPC −2.57% [95% CI: −3.14% to −2.11%] vs 2010 to 2020 AAPC −1.27% [95% CI: −1.93% to −0.85%]) and noncore (1999-2009 AAPC of −2.86% [95% CI: −3.27% to −2.41%] vs 2010 to 2020 AAPC −1.77% [95% CI: −2.70% to −0.87%]) had stalled in comparison to urban centers.

We found an overall decline in mortality rates in the general population and across sex, race, census regions, and urbanization strata during the 22-year period. However, there remained persistent sex and racial disparities in the rate of NICM deaths. Mortality continued to be highest among African Americans compared to other race groups and higher in males compared to females. Strikingly, between 2010 and 2020, the decline in NICM deaths stalled in AI/AN population with a nominal decrease of 0.49%. A similar less rate of decline in mortality rate was also seen in the Midwest, West, and nonurban areas. These findings suggest that despite advances in guideline-directed medical therapies and advanced therapies for cardiogenic shock or stage D cardiomyopathy, segments of the population may not derive the same benefit.

Our data highlight persistent disparities and gaps in care in the management of NICM. It is recognized that adults who belong to underrepresented racial and ethnic groups confront more barriers to cardiovascular disease diagnosis and care, receive less optimal care, and experience worse adverse outcomes than White adults.^6^ Previous data have highlighted the contemporary trends in adult heart transplant outcomes among individuals from different racial and ethnic groups.^7^^,^^8^ Compared with White adults, Black adults were still less likely to undergo transplantation and had a higher risk of post-transplant death despite recent amendments to the United Network for Organ Sharing.^9^ These disparities may be driven by factors such as structural racism, bias, stereotyping, and prejudice against underrepresented minority populations.^9^^,^^10^ The differential access to quality health care is pervasive and occurs in the broader context of historic and contemporary social and economic practices and policies that have led to inequalities across many U.S. health care systems.^10^ Of note, the deceleration in the rate of decline observed among AI/AN may indicate inadequately resourced health systems and other challenges related to social determinants of health unique to this population.

Utilizing a national database strengthens the generalizability of our findings. However, the study has some limitations. Firstly, this is an observational study, and as such, it does not establish causality but only informs association and residual confounding cannot be excluded. Secondly, the data used for this study were collected from death certificates, which have the potential for misclassification bias. Thirdly, the lack of patient-level data and data on the utilization of guideline-directed medical therapy, left ventricular assist device implantation, and heart transplantation precludes adjustment for confounders.

In conclusion, our findings provide evidence in support of the persistent sex, racial, rural-urban, and geographical inequities in NICM outcomes and underscore an unmet need for targeted clinical and policy-level efforts to address these gaps.
